# Supplementary material for: A Novel Strategy for Regulating mRNA’s Degradation via Interfering the AUF1’s Binding to mRNA
Source: Molecules. 2022 May 16;27(10):3182. doi: 10.3390/molecules27103182 (PMC9143527; doi:10.3390/molecules27103182)
Supplement: Supplementary file 1 [file molecules-27-03182-s001.zip › molecules-1706317-supplementary.pdf]

molecules

Supplementary Information

A novel strategy for regulating mRNA's degradation via interfering the AUF1's binding to mRNA

Kun-Tao Li, Xiong-Zhi Wu, Zhi-Yin Sun and Tian-Miao Ou*

School of Pharmaceutical Sciences, Sun Yat-sen University, Guangzhou 510006, China; 1596187273@qq.com (L.-K.T.); wuxzh9@mail.sysu.edu.cn (W.-X.Z.); sunzhy0118@163.com (S.-Z.Y.);
* Correspondence: Correspondence: outianm@mail.sysu.edu.cn; Tel.: +86-20-39943055

CONTENT

1. Supplementary Methods……………………………………………..1
2. Supplementary Tables…………………………………………….……4
3. Supplementary Figures……………………………………………..19

Supplementary Methods

Chemistry. All chemicals used for synthesis and biological experiments were purchased from commercial suppliers unless specified. All chemical structures were confirmed by ^{1}H and ^{13}C NMR spectra and high-resolution mass spectra (HRMS) spectrometry. ^{1}H and ^{13}C NMR spectra were measured using TMS as the internal standard in DMSO-d_6, CD_3OD, or $CDCl_3$ on a Bruker BioSpin GmbH spectrometer at 400 MHz and 500 MHz, respectively. HRMS were obtained on a MAT95XP mass spectrometer (ThermoFisher Scientific). The purity of the synthesized compounds was ensured to be above 95% by using an analytical HPLC performed with a dual pump Shimadzu LC-20 AB system equipped with an Ultimate XB-C18 column (4.6 × 250 mm, 5 μm) and eluted with a methanol-water (35:65-80:20) containing 0.1% TFA at a flow rate of 0.5 mL/min or 0.2 mL/min.

*Synthesis of diamino triazole (**d**).* A solution of aniline **b** (1.20 mmol) in isopropanol (4 mL) was treated with diphenyl N-cyanocarbonimidate (1.00 mmol). The reaction mixture was maintained at room temperature for 16 h, filtered, and rinsed with ethyl ether (5 mL). The precipitate was suspended in EtOH (10 mL), treated with hydrazine hydrate (0.2 ml, 3.3 mmol), and heated to reflux for 2.5 h. Compound d precipitated from the reaction mixture and was filtered, washed with ethyl ether (2 × 5 mL) and dried under vacuum.

*(5-amino-3-((4-(tert-butyl)phenyl)amino)-1H-1,2,4-triazol-1-yl)(4-(trifluoromethyl) phenyl)methanone (**1**).* A solution of triazole **29d** (274 mg, 1.2 mmol) in pyridine (1.5 mL) was treated with 4-(trifluoromethyl)-benzoylchlorid (226 μL, 1.5 mmol). The reaction mixture was maintained at room temperature for 24 h, concentrated, and purified by silica gel column chromatography using DCM/EtOAc (20:1, v/v) as mobile phase, yielding the title compound as a yellow solid (yield 42.0%); m.p.: 186–188 °C. **^{1}H NMR** (400 MHz, DMSO) δ (ppm) 9.19 (s, 1H), 8.33 (d, J = 8.1 Hz, 2H), 7.96 (d, J = 8.2 Hz, 2H), 7.85 (s, 2H), 7.40 (d, J = 8.6 Hz, 2H), 7.23 (d, J = 8.5 Hz, 2H), 1.23 (s, 9H). **^{13}C NMR** (126 MHz, DMSO) δ (ppm) 165.2, 158.6, 157.3, 142.4, 138.2, 136.7, 131.7, 131.0, 125.3, 124.9, 122.7, 116.4, 33.8, 31.3. **^{19}F NMR** (376 MHz, DMSO) δ (ppm) -61.4. HRMS (TOF MS ESI$^+$) m/z: $[M + H]^+$ calculated for $C_{20}H_{20}F_3N_5O$ 404.1693; found 404.1692.

*N-(4-((5-amino-1-(2,6-difluorobenzoyl)-1H-1,2,4-triazol-3-yl)amino)phenyl) acetamide (**2**).* A solution of triazole **30d** (200 mg, 0.9 mmol) in pyridine (1.5 mL) was treated with 2,6-difluorobenzoyl chloride (133 μL, 1.2 mmol). The reaction mixture was maintained at room temperature for 24 h, concentrated, and purified by silica gel column

chromatography using DCM/MeOH (20:1, v/v) as mobile phase, yielding the title compound as a white solid (yield 25.0%); m.p.: 230–231 °C. **^{1}H NMR** (400 MHz, DMSO) δ (ppm) 9.68 (s, 1H), 9.23 (s, 1H), 7.88 (s, 2H), 7.74 – 7.64 (m, 1H), 7.37 – 7.28 (m, 4H), 7.24 (d, J = 9.0 Hz, 2H), 1.97 (s, 3H). **^{13}C NMR** (101 MHz, DMSO) δ (ppm) 167.7, 159.7 (d, J = 7.2 Hz), 159.2, 159.0, 157.2 (d, J = 7.3 Hz), 156.2, 136.2, 133.3 (t, J = 9.9 Hz), 132.4, 119.6, 117.0, 112.9 (t, J = 21.6 Hz), 112.1 (d, J = 4.1 Hz), 111.9 (d, J = 3.9 Hz), 23.8. **^{19}F NMR** (471 MHz, DMSO) δ (ppm) -112.8 (t, J = 7.3 Hz). HRMS (TOF MS ESI^{+}) m/z: [M + Na]$^{+}$ calculated for $C_{17}H_{14}F_2N_6O_2$ 395.1039; found 395.1047.

N-(4-((5-amino-1-(4-(trifluoromethyl)benzoyl)-1H-1,2,4-triazol-3-yl)amino)phenyl) acetamide (3). A solution of triazole **32d** (264 mg, 1.1 mmol) in pyridine (1.5 mL) was treated with 4-(trifluoromethyl)-benzoylchlorid (226 μL, 1.5 mmol). The reaction mixture was maintained at room temperature for 24 h, concentrated, and purified by silica gel column chromatography using DCM/MeOH (20:1, v/v) as mobile phase, yielding the title compound as a yellow solid (yield 22.2%); m.p.: 390–391 °C. **^{1}H NMR** (400 MHz, DMSO) δ (ppm) 9.71 (s, 1H), 9.22 (s, 1H), 8.29 (d, J = 8.1 Hz, 2H), 7.95 (d, J = 8.2 Hz, 2H), 7.84 (s, 2H), 7.39 (s, 4H), 1.98 (s, 3H). **^{13}C NMR** (126 MHz, DMSO) δ (ppm) 167.7, 165.3, 158.5, 157.3, 136.8, 136.4, 132.4, 131.0, 130.1, 124.8, 124.8, 122.7, 119.8, 116.9, 23.8. **^{19}F NMR** (471 MHz, DMSO) δ (ppm) -61.5. HRMS (TOF MS ESI^{+}) m/z: [M + H]$^{+}$ calculated for $C_{18}H_{15}F_3N_6O_2$ 405.1281; found 405.1281.

Construction, expression, and purification of AUF1 protein. The cDNA encoding p37^{AUF1} was subcloned into pGEX-4T-1 vector for generation recombinant protein in BL21(DE3) *E. coli* cells. An N-terminal GST peptide was attached for purification by Glutathione Sepharose 4B column (GSTrap 4B; GE Healthcare). The purification of p37^{AUF1} was conducted essentially as previously described reference [26]. The protein was concentrated with Amicon Ultra centrifugal filters (Merck millipore), and the elution buffer was replaced with 10mM Tris-HCl (pH 7.4). The protein concentration was determined by Pierce™ BCA Protein Assay Kit (ThermoFisher).

MTT assay. The cells were seeded on 96-well plates (5,000 cells/well) and treated with various concentrations of test compounds. After 48-h treatment, 20 μL of 2.5 mg/mL 3-(4,5-dimethyl-2-thiazolyl)-2,5-diphenyl-2-H-tetrazolium bromide (MTT) solution was added to each well and further incubated for four hours. Cells in each well were then treated with dimethyl sulfoxide (DMSO) (100 μL for each well), and the optical density (OD) was recorded at 570 nm. Statistical analysis of IC50 values were calculated from concentration-response curves by GraphPad Prism 7.0 software (GraphPad Software, San Diego, CA, USA), using a sigmoidal model equation.

Reporter plasmid construction. The whole sequence from the 3′-UTR of IL8 was synthesized and cloned into a pCpGfree-basic-Lucia vector (InvivoGen). The 3′del luciferase reporter was constructed by generating the sequence from the 3′WT plasmid by using PCR amplifying and standard cloning procedure. All the constructs were confirmed by DNA sequencing.

Table S1. The RU values of 186 selected compounds.

compounds	RU	compounds	RU	compounds	RU
3586-A2	39.6	3597-H5	74.1	3604-F4	27.9
3586-B4	75.2	3597-H11	41.3	3604-F5	30.1
3586-B6	51.7	3598-B10	52.0	3604-F6	144.1
3586-F2	79.1	3598-F2	37.7	3604-F7	26.7
3586-F3	50.7	3598-F9	25.8	3604-F8	76.9
3587-A8	47.4	3598-G5	102.2	3604-F10	32.8
3587-A11	49.1	3598-G6	69.1	3604-G5	28.9
3587-E2	31.0	3598-G11	66.4	3604-G7	25.2
3587-E3	53.4	3598-H2	47.0	3604-G9	25.3
3587-E5	153.0	3598-H8	39.6	3604-G10	26.0
3587-E7	33.0	3599-B10	27.8	3604-G11	31.8
3587-E8	68.0	3599-C5	120.6	3604-H2	24.3
3587-E9	20.7	3599-C6	43.6	3604-H4	31.2

3587-E10	58.1	3599-C7	20.6	3604-H5	20.2
3587-E11	32.0	3599-D11	20.5	3604-H6	24.4
3587-F7	36.0	3599-E10	69.9	3604-H7	23.5
3587-F11	31.5	3599-F2	29.1	3604-H9	24.8
3587-G8	31.6	3599-H5	25.6	3604-H10	25.9
3587-G11	28.2	3599-H7	24.0	3605-B11	72.8
3587-H5	31.4	3599-H10	34.8	3605-D4	21.8
3587-H8	32.4	3600-A2	30.7	3605-D6	40.7
3587-H10	31.2	3600-A7	56.6	3605-D8	70.1
3588-E7	37.3	3600-A9	31.0	3605-D9	33.4
3588-E8	30.0	3600-D4	27.7	3605-D11	135.2
3588-E11	26.7	3600-F5	57.8	3605-F10	83.7
3588-F7	34.4	3600-F8	49.8	3605-H9	34.8
3588-G4	38.1	3600-G8	22.9	3606-B11	31.8
3589-D4	28.6	3600-H7	70.2	3606-C10	26.8
3589-E9	20.7	3601-A6	84.9	3606-F6	64.1
3589-E10	58.1	3601-C3	46.2	3606-G3	47.6
3589-E11	38.2	3601-D6	72.2	3606-G5	23.2
3589-H3	80.8	3601-D10	21.4	3606-G6	108.0
3590-D4	31.6	3601-D11	52.2	3606-G8	25.2
3590-D7	22.4	3601-F3	31.3	3606-H9	35.2
3590-H11	69.6	3601-H2	127.9	3607-C4	23.7
3591-D8	22.7	3601-H3	32.1	3607-D11	109.9
3592-D3	51.5	3601-H7	118.7	3607-E11	120.8
3592-D8	26.2	3601-H8	33.4	3607-F9	40.6
3593-D9	62.8	3601-H10	150.7	3607-G10	53.5
3593-F2	20.2	3602-B5	48.8	3607-H7	43.4
3593-H2	38.3	3602-B8	84.6	3608-A2	78.0
3593-H4	34.5	3602-B11	41.3	3608-A10	84.5
3594-B7	28.9	3602-D5	87.0	3608-A11	115.7
3594-C3	37.4	3602-D7	72.4	3608-E11	52.6
3594-F7	32.6	3602-D8	132.1	3608-F5	133.4
3595-A8	45.7	3602-D9	127.8	3608-F6	93.1
3595-B4	29.3	3602-D11	55.7	3608-F7	35.2
3595-B10	36.0	3602-E4	57.0	3608-F2	40.1
3595-E7	22.9	3602-E9	41.4	3608-F4	86.2
3595-E8	30.8	3602-F2	78.7	3608-D8	21.4
3595-F3	30.8	3602-F7	42.6	3609-A3	32.5
3595-F6	26.0	3602-H7	23.7	3609-C10	31.5
3596-A3	33.2	3602-H9	25.8	3610-A3	20.2
3596-A5	51.0	3603-D7	37.0	3610-A5	23.4
3596-B6	116.7	3603-D8	33.0	3610-A6	20.4
3596-E5	43.4	3603-D10	36.9	3610-A9	45.3
3596-F10	46.8	3603-D11	94.2	3609-A11	20.0
3596-H5	31.0	3603-F6	24.2	3609-B4	22.8
3597-B7	64.5	3603-H6	85.0	3609-B10	39.7
3597-B8	47.8	3604-B9	131.3	3609-D2	35.6
3597-F9	104.4	3604-E11	25.4	3609-D3	51.5
3597-G3	40.5	3604-F3	44.4	3610-A4	59.8

Table S2. Sequence of wild-type and deletion 3′UTR of *IL8* mRNA in dual-luciferase reporter construction.

**wild-type 3'-UTR (3'WT), 5'-3'**
AAAAATTCATTCTCTGTGGTATCCAAGAATCAGTGAAGATGCCAGTGAAACTTCAA-GCAAATCTACTTCAACACTTCATGTATTGTGTGGGTCTGTTGTAGGGTTGCCAGATGCAATACA AGATTCCTGGTTAAATTTGAATTTCAGTAAACAATGAATAGTTTTTCATTGTACCATGAAA-TATCCAGAACATACTTATATGTAAAGTATTATTTATTTGAATCTACAAAAAACAACAAATAATT TTTAAATATAAGGATTTTCCTAGATATTGCACGGGAGAATATACAAATAGCAAAATT-GAGGCCAAGGGCCAAGAGAATATCCGAACTTTAATTTCAGGAATTGAATGGGTTTGCTAGAAT GTGATATTTGAAGCATCACATAAAAATGATGGGACAATAAATTTTGCCA-TAAAGTCAAATTTAGCTGGAAATCCTGGATTTTTTTCTGTTAAATCTGGCAACCCTAGTCTGCTA GCCAGGATCCACAAGTCCTTGTTCCACTGTGCCTTGGTTTCTCCTTTATTTCTAAGTG-GAAAAAGTATTAGCCACCATCTTACCTCACAGTGATGTTGTGAGGACATGTGGAAGCACTTTA AGTTTTTTCATCATAACATAAATTATTTTCAAGTGTAACTTATTAAC-CTATTTATTATTTATGTATTTATTTAAGCATCAAATATTTGTGCAAGAATTTGGAAAAATAGAA

GATGAATCATTGATTGAATAGTTATAAAGATGTTATAGTAAATTTATTTTATTTTAGA-<br>TATTAAATGATGTTTTATTAGATAAATTTCAATCAGGGTTTTTAGATTAAACAAACAAACAATT<br>GGGTACCCAGTTAAATTTTCATTTCAGATAAACAACAAATAATTTTTTAG-<br>TATAAGTACATTATTGTTTATCTGAAATTTTAATTGAACTAACAATCCTAGTTTGATACTCCCAG<br>TCTTGTCATTGCCAGCTGTGTTGGTAGTGCTGTGTTGAATTACGGAATAATGAGTTAGAAC-<br>TATTAAAACAGCCAAAACTCCACAGTCAATATTAGTAATTTCTTGCTGGTTGAAACTTGTTTAT<br>TATGTACAAATAGATTCTTATAATATTATTTAAATGACTGCATTTTTAAATACAAGGCTTTA-<br>TATTTTTAACTTTAAGATGTTTTTATGTGCTCTCCAAATTTTTTTTACTGTTTCTGATTGTATGGA<br>AATATAAAAGTAAATATGAAACATTTAAAATATAATTTGTTGTCAAAGTAA
**deletion of ARE in 3’-UTR (3’del), 5’-3’**
AAAAATTCATTCTCTGTGGTATCCAAGAATCAGTGAAGATGCCAGTGAAACTTCAA-<br>GCAAATCTACTTCAACACTTCATGTATTGTGTGGGTCTGTTGTAGGGTTGCCAGATGCAATACA<br>AGATTCCTGGTTAAATTTGAATTTCAGTAAACAATGAATAGTTTTTCATTGTACCATGAAA-<br>TATCCAGAACATACTTATATGTAAAGTATTATTTATTTGAATCTACAAAAAACAACAAATAATT<br>TTTAAATATAAGGATTTTCCTAGATATTGCACGGGAGAATATACAAATAGCAAAATT-<br>GAGGCCAAGGGCCAAGAGAATATCCGAACTTTAATTTCAGGAATTGAATGGGTTTGCTAGAAT<br>GTGATATTTGAAGCATCACATAAAAATGATGGGACAATAAATTTTGCCA-<br>TAAAGTCAAATTTAGCTGGAAATCCTGGATTTTTTTCTGTTAAATCTGGCAACCCTAGTCTGCTA<br>GCCAGGATCCACAAGTCCTTGTTCCACTGTGCCTTGGTTTCTCCTTTATTTCTAAGTG-<br>GAAAAAGTATTAGCCACCATCTTACCTCACAGTGATGTTGTGAGGACATGTGGAAGCACTTTA<br>AGTTTTTTCATCATAACATAAATTATTTTCAAGTGTAACTTATAAATATTTGTGCAA-<br>GAATTTGGAAAAATAGAAGATGAATCATTGATTGAATAGTTATAAAGATGTTATAGTAAATTT<br>ATTTTATTTTAGATATTAAATGATGTTTTATTAGATAAATTTCAATCAGGGTTTTTAGAT-<br>TAAACAAACAAACAATTGGGTACCCAGTTAAATTTTCATTTCAGATAAACAACAAATAATTTTT<br>TAGTATAAGTACATTATTGTTTATCTGAAATTTTAATTGAACTAACAATCCTAGTTTGA-<br>TACTCCCAGTCTTGTCATTGCCAGCTGTGTTGGTAGTGCTGTGTTGAATTACGGAATAATGAGT<br>TAGAACTATTAAAACAGCCAAAACTCCACAGTCAATATTAGTAATTTCTTGCTGGTT-<br>GAAACTTGTTTATTATGTACAAATAGATTCTTATAATATTATTTAAATGACTGCATTTTTAAATA<br>CAAGGCTTTATATTTTTAACTTTAA-<br>GATGTTTTTATGTGCTCTCCAAATTTTTTTTACTGTTTCTGATTGTATGGAAATATAAAAGTAAA<br>TATGAAACATTTAAAATATAATTTGTTGTCAAAGTAA

Table S3. Primers used in qRT-PCR.

Primer	Forward (5’-3’)	Reverse (5’-3’)
AUF1	GATCAAGGGGTTTTGGCTTT	GTTGTCCATGGGGACCTCTA
EMP3	CGAGAATGGCTGGCTGAAG	GCCACGCTGGTGCAAAG
IL8	CACTGCGCCAACACAGAAAT	GCCCTCTTCAAAAACTTCTCCAC
PLEK2	TGGAGTTAAGTGGCACGGTG	GAGCAGACACGAGTGAACCA
BTG2	GCGTGAGCGAGCAGAGGCTT	GGCTGGCCACCCTGCTGATG
β-Actin	TCCCTGGAGAAGAGCTACGA	AGGAAGGAAGGCTGGAAGAG

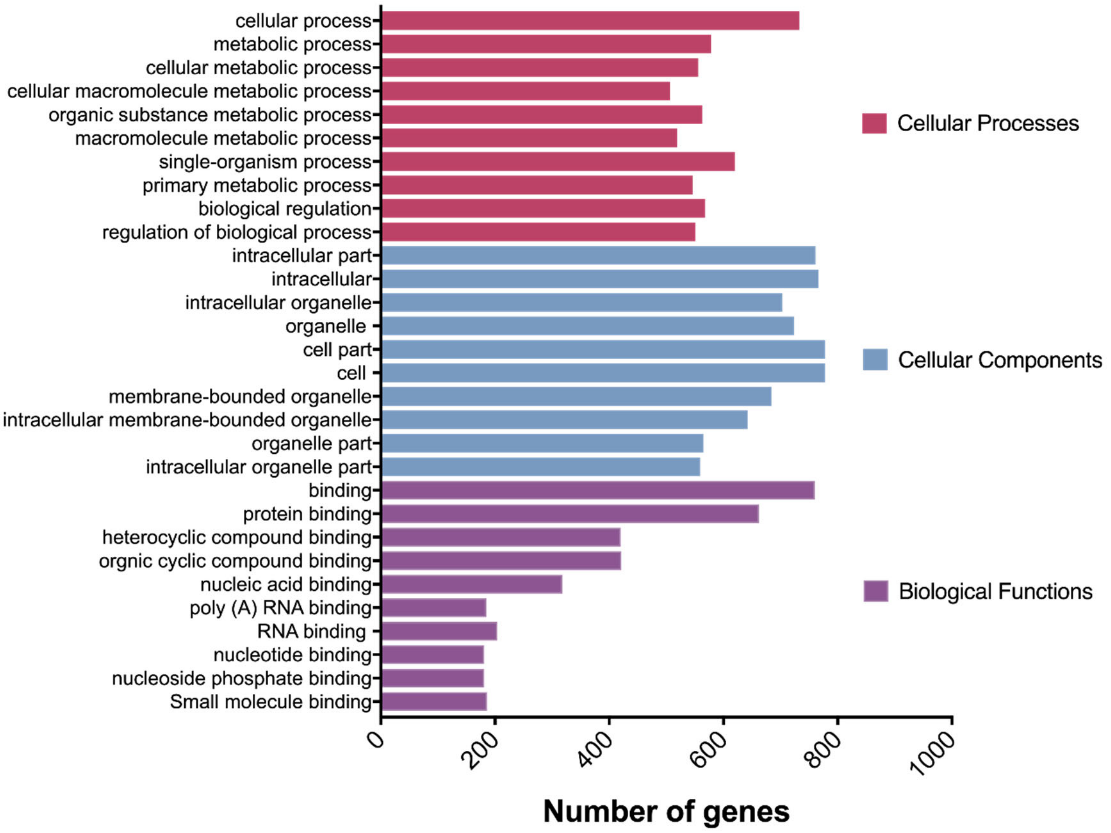

Figure S1. Gene ontology (GO) of AUF1 target genes. The significance threshold of P<0.05 was used to obtain statistically significant high-frequency annotation for the distribution information and significance of candidate genes in different GO categories. The X-axis shows the number of genes, and the left Y-axis shows the relevant biological functions. Pink columns represent cellular processes, blue columns represent involved cellular components, and violet columns represent biological functions.

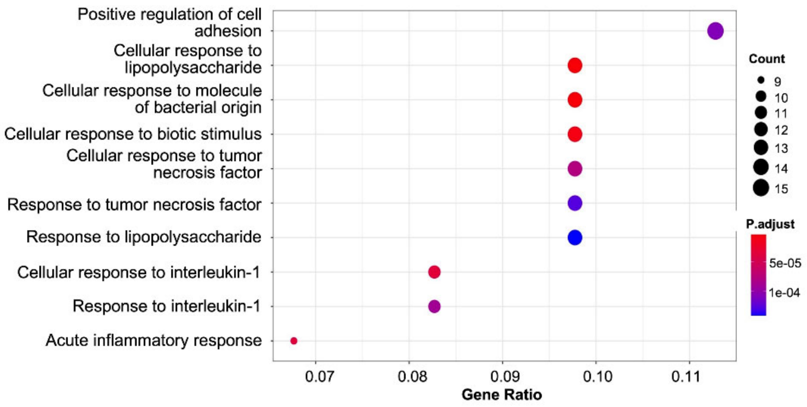

Figure S2. Gene ontology (GO) of JNJ-7706621 regulated genes. The significance threshold of $P<0.05$ was used to obtain statistically significant high-frequency annotation for the distribution information and significance of candidate genes in different GO categories.

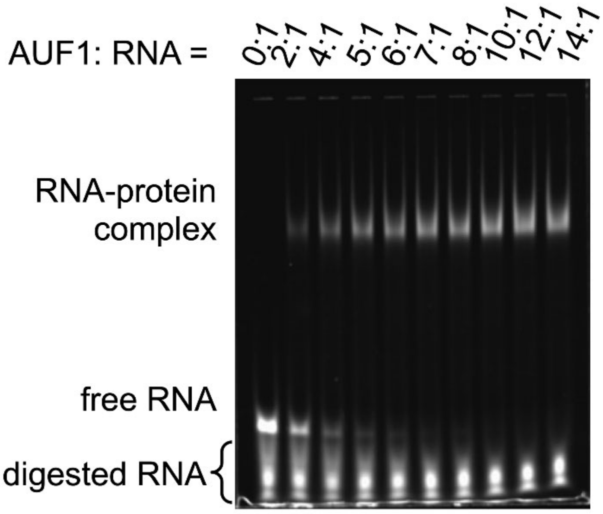

Figure S3. The REMSA result of the FAM-labelled RNA (250 nM) incubated with AUF1 at increasing concentrations. The sample was incubated in an incubation buffer (10 mM Tris-HCl, pH 8.0, containing 50 mM KCl, 2 mM DTT, 0.5 mM EDTA, and 0.1 μg/μl BSA) and loaded on a 6% native polyacrylamide gel.

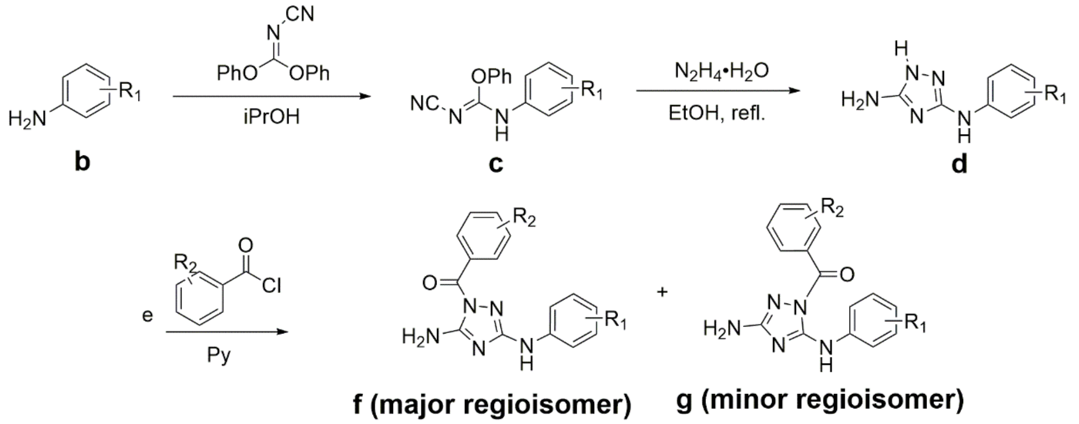

Figure S4. General synthesis of 1,2,4-triazole-3,5-diamine derivatives. Treatment of aniline **b** with diphenyl N-cyanocarbonimidate afforded the aniline adduct **c**, which was heated to reflux with hydrazine hydrate to give the diaminotriazole **d**. Reaction of d with benzoyl chloride **e** gave a mixture of regioisomers **f** (major) and **g** (minor).
